# Illuminating the Neural Circuits Underlying Orienting of Attention

**DOI:** 10.3390/vision3010004

**Published:** 2019-01-24

**Authors:** Michael I. Posner, Cristopher M. Niell

**Affiliations:** 1 Institute of Neuroscience, University of Oregon, Eugene, OR 97401, USA; 2 Department of Psychology, University of Oregon, Eugene, OR 97403, USA; 3 Department of Biology, University of Oregon, Eugene, OR 97401, USA

**Keywords:** attention networks, magnocellular and parvocellular pathways, optogenetics, orienting

## Abstract

Human neuroimaging has revealed brain networks involving frontal and parietal cortical areas as well as subcortical areas, including the superior colliculus and pulvinar, which are involved in orienting to sensory stimuli. Because accumulating evidence points to similarities between both overt and covert orienting in humans and other animals, we propose that it is now feasible, using animal models, to move beyond these large-scale networks to address the local networks and cell types that mediate orienting of attention. In this opinion piece, we discuss optogenetic and related methods for testing the pathways involved, and obstacles to carrying out such tests in rodent and monkey populations.

## 1. Introduction

Orienting toward a stimulus or location has been an important model for the study of attention and has also been used as a common task to compare humans, monkeys, and rodents. The source of the orienting effect is a brain network described in the next section, which serves to amplify information coming from sites in the visual, auditory, or somatosensory cortex. The orienting network is distinct from networks involved in the alert state and in controlling voluntary response output [1]. In this paper, we attempt to build upon the study of brain networks involved in orienting of attention. We discuss how controlling the activity of neurons can be used to promote further integration across different levels, from neurons up to networks and brain function.

A model task, using visual cues, is appropriate for both humans and non-human animals, to study simple orienting in an empty visual field [2]. This task used cues occurring at the location of a likely target (exogenous cuing) and those presented at fixation that provide a basis for a voluntary movement of attention to the target location (endogenous cuing). This task, designed to study covert orienting, has the following properties: (1) It presents a cue to direct attention to a target location; (2) it monitors eye movements to eliminate overt shifts; (3) it uses a single detection response to avoid differential muscular preparation; and (4) it measures reaction time to respond to the target to assay improvements due to the cue.

## 2. Brain Networks of Orienting

In the era of neuroimaging, it has become possible to trace the neuronal networks involved in orienting of attention in humans. An important summary of this work [3] showed that the parietal areas involved in voluntary orienting in response to central arrow cues involved a dorsal pathway through the superior parietal lobe. The superior parietal lobe appears to be crucial in orchestrating a voluntary shift of attention by a cue, which precedes a target. This brain area is closely tied with voluntary eye movements that involve the frontal eye fields, as would be predicted by the premotor theory of attention shifts. However, a different, more ventral area of the parietal lobe, the temporal parietal junction (TPJ) is the area involved in orienting to a peripheral event, for example, to a stimulus at an unexpected location that acts to summon orienting. The TPJ influence on orienting seems to be strongly lateralized to the right hemisphere, while the voluntary shift appears to be more bilateral [3]. Both of these pathways through the parietal lobe are distinct from the “what” pathway involving the inferior temporal lobe discussed below. In addition to these cortical areas, human and animal studies have suggested involvement of subcortical areas, including the superior colliculus [4] and pulvinar [5] in the orienting network.

A recent paper from Lambert et al. [6] identified two forms of orienting of attention. One form, landmark cuing, involves a rapid shift and uses the dorsal pathway through the parietal lobe. Cues such as symmetric letters, when remote from the target, use a slower ventral “what pathway” [7] through occipital and temporal lobes. Lambert et al. [6] had no imaging data but argued for the ventral route from behavioral data showing that the symbolic cues had temporal properties consonant with the parvocellular neurons related to the “what” pathway. On the other hand, landmark orienting was more consistent with magnocellular neurons that are related to the parietal pathway. Lambert et al. [6] argued that Corbetta and Shulman [3] confounded cuing results using asymmetric cues, like arrows, which could produce reflexive orienting. However, Lambert et al. [6] confounded symmetric symbolic cues with letters, so it is not certain if the ventral orienting pathway is true of all symbolic symmetric cues or whether it is unique to letter or letter-like stimuli. In the supplement to their article, central number cues, sometimes symmetric and sometimes asymmetric, were presented. When the presentation interval was brief, only the asymmetric cue could lead to successful orienting. The authors argued this was because asymmetric cues could use the faster dorsal pathway. The authors argued that number cues were related to properties of parvocellular stimuli because of their allowing successful cuing with very brief exposure durations. Overall, Lambert et al. [6] have mainly shown the ventral cortex is active for letter stimuli, which, as is known from previous work [8] on the visual word form system, involves the left ventral cortex and, thus, the parvocellular pathway. We believe that Lambert [6], using letter-like stimuli to cue orienting, confounded orienting with identification of the cue by the visual word-form system.

Thus, three cortical pathways have been reported as being involved in the orienting of attention: Both dorsal (superior parietal cortex) and ventral (temporal-parietal junction) parietal pathways and an even more ventral temporal lobe route related to the “what” pathway. It remains to be shown which pathways are actually involved when cues are not confounded with symmetry and linguistic factors.

## 3. Orienting in Animal Behavior

**Figure 1:**
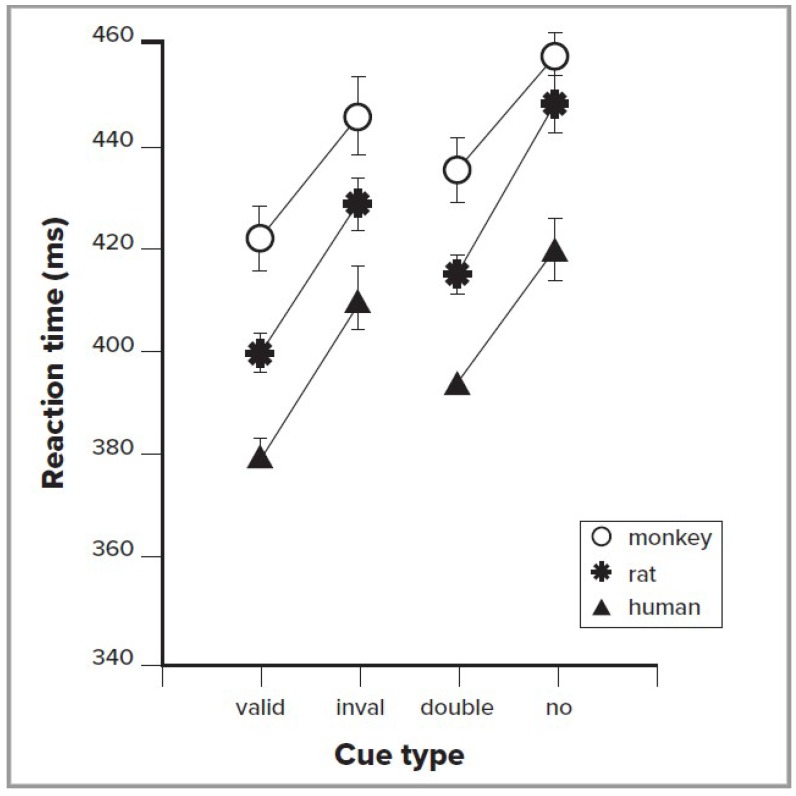
Reaction times (RT) for three species. The left side of the figure compares when the cue correctly indicates the location of the target (valid), versus when it indicates an incorrect target location (invalid). Subtracting valid RT from invalid RT gives the time to reorient to the location of target. The right side of the figure indicates reaction time when the cue indicates when, but not where, a target will occur (double), versus when the target is not cued (none). The subtraction between these produces the alerting score. Adapted with permission from Beane and Marrocco [9].

A number of primate and other animal studies have also used the cuing method. These studies include behavioral measures such as reaction time, as well as recording from individual neurons in various brain regions. Behavioral data, using the simple exogenous cuing method, have shown impressive convergence across human, monkey, and rodent studies, as illustrated in Figure 1. The left side of this figure shows the reaction time to valid exogenous cues, which indicate the target location 80% of the time, and invalid cues, which indicate the correct target location only 20% of the time [9]. It is very clear from these data that the three species all improve in RT with valid cues. The right side of the figure compares a double cue, which provides no spatial information, with a no cue condition, to allow measurement of the alerting effect. The alerting effect also is similar across species.

Recently, a new behavioral paradigm was developed to probe spatially selective attention in head-fixed mice [10], using a go/no-go change detection task. The mouse received a vertical grating cue in either the left or right visual field (or no cue), followed by presentation of vertical targets in both fields. The subject then had to detect a change in orientation of one of the targets after a random interval, responding by licking. This study showed that mice can perform orienting of spatial attention in a similar manner shown for other species in Figure 1, resulting in improved accuracy and reduced reaction time for validly cued stimuli. This demonstration of a paradigm for the orienting of attention in mice opens the possibility of applying genetic approaches to manipulate neural activity, in order to causally test neural circuit mechanisms.

Research suggests that there may be both temporal and spatial flexibility in the operation of a spotlight model often used to account for spatial orienting effects. The idea of a fixed spotlight, covertly orienting to a spatial location, was modified by studies showing that orienting can adjust to the spatial frequency of the target when presented with mixed large and small targets. Navon [11] constructed a large letter made of small letters and found that orienting could adjust to the letter size by showing a cuing effect based on size. Furthermore, recent data also suggest that orienting may not be continuous in time. Monkeys and humans show higher accuracy detections at particular phases of a 4 Hz rhythm, with corresponding neural correlates, suggesting the orienting is not constant but varies over time [12,13,14].

## 4. Consequences of Orienting on Visual Processing

How does successful orienting of attention influence processing of visual targets? Figure 1 shows that when a valid cue is used to orient attention, reaction time is improved in comparison to when an invalid cue forces reorienting attention. This suggests that attention provides priority of processing for a visual signal.

Efforts in humans to examine the electrical potential evoked by a stimulus showed that attended areas also have larger response amplitudes than other areas of the visual field [15]. This finding of a higher amplitude response to attended stimuli has also been found in a number of studies using single-neuron responses in non-human primates [16], along with other effects, such as changes in neural correlations [17,18].

**Figure 2:**
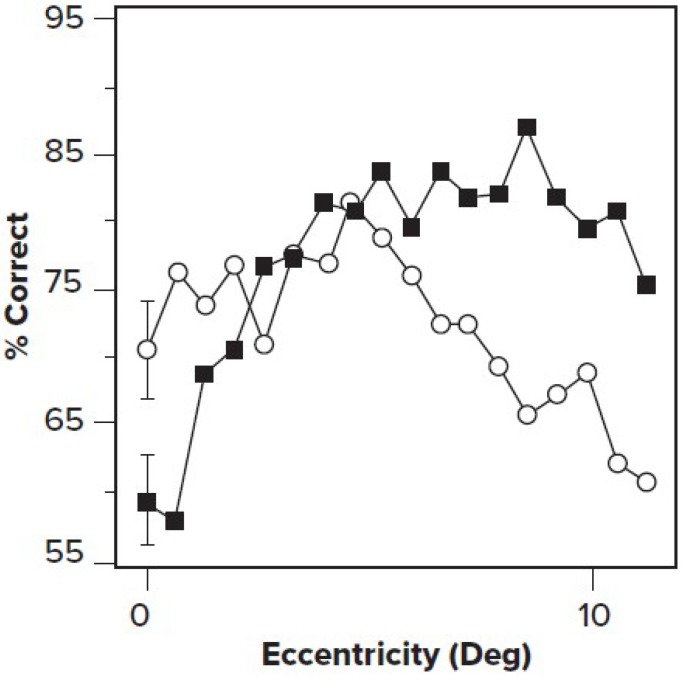
Cued stimulation (solid squares) results in a higher percentage correct than uncued (open circles) in the periphery (high eccentricity), but near to the fovea (low eccentricity), performance on uncued targets (open circles) is better. Reaction times did not differ. Adapted from Yeshurun and Carrasco [19] with permission.

In a brilliant series of experiments, Yeshurun and Carrasco [19], using the cueing method described above, coupled with sinusoidal grating targets, showed that attention improved visibility for high-spatial-frequency information presented in the periphery while reducing accuracy for foveal stimuli. Carrasco [20] argued that in the fovea, where spatial frequency resolution was higher than optimal, attention actually impaired performance, while at the periphery, where spatial resolution was low, attention improved performance (See Figure 2).

## 5. Genetic-Based Manipulations of Neural Activity

Over the past fifteen years, a range of methods have been developed to manipulate neural activity using genetically encoded effectors. Optogenetics uses channels or other proteins that can be activated by light to depolarize or hyperpolarize cells [21]. Chemogenetics uses receptors that are modified to bind exogenous ligands [22]. Together, these provide powerful approaches to turn on and off specific pathways and cell types in the intact brain. Can these methods be used to illuminate networks important for orienting of attention?

The orienting network has widespread cortical and subcortical nodes, requiring precise patterns and timing of connectivity. We have been using optogenetics in mice to test the hypothesis that rhythmic neural activity influences the connectivity of specific brain networks involved in attention. This work is inspired by our previous findings in humans, using a form of mindfulness meditation [23] called Integrated mind–body training (IBMT). We randomized a group of undergraduates either to 5 days of IBMT or to a relaxation active control condition. As expected, we obtained improvements in attention, mood, and reduced stress in IBMT compared to the active control. However, surprisingly, we found that 2–4 weeks of IBMT training, compared to controls, improved white matter surrounding the anterior cingulate as measured by a positive change in fractional anisotropy (FA) [24]. Subsequently [25], it was found that after two weeks, the FA change seemed to involve primarily changes in axial diffusivity (likely corresponding to axonal size or density), while after 4 weeks, we also found evidence of decreased radial diffusivity (corresponding to increased myelin).

At that time, it was surprising that white matter should change in adults following meditation training. However, in the last few years, many adult human and animal studies have shown improvements in white matter with training [26,27] and with direct stimulation of neural activity [28]. How might a purely mental practice like IBMT produce a change in white matter? We suggested [29] that the frontal theta (4–8 Hz), which was found to be increased even at rest following two weeks of meditation training [30], might induce myelination by activating dormant oligodendrocytes through activity-dependent mechanisms.

To test the possible role of oscillatory activity in myelination, we used an optogenetic approach in mice in order to drive rhythmic activity in anterior cingulate cortex (ACC) [31,32]. Specifically, we used a parvalbumin (PV) Cre driver line, which expresses in a subset of inhibitory neurons, together with a Cre-dependent Archaerhodopsin-2 (Arch) line to provide cell type-specific reduction of inhibitory activity. We then implanted laser-coupled optical fibers to increase the firing output of cells in the ACC in rhythm with the light stimulation. Stimulation was provided via rhythmic laser input at 1, 8, or 40 Hz, or no stimulation, for twenty sessions of half an hour each over a month.

**Figure 3:**
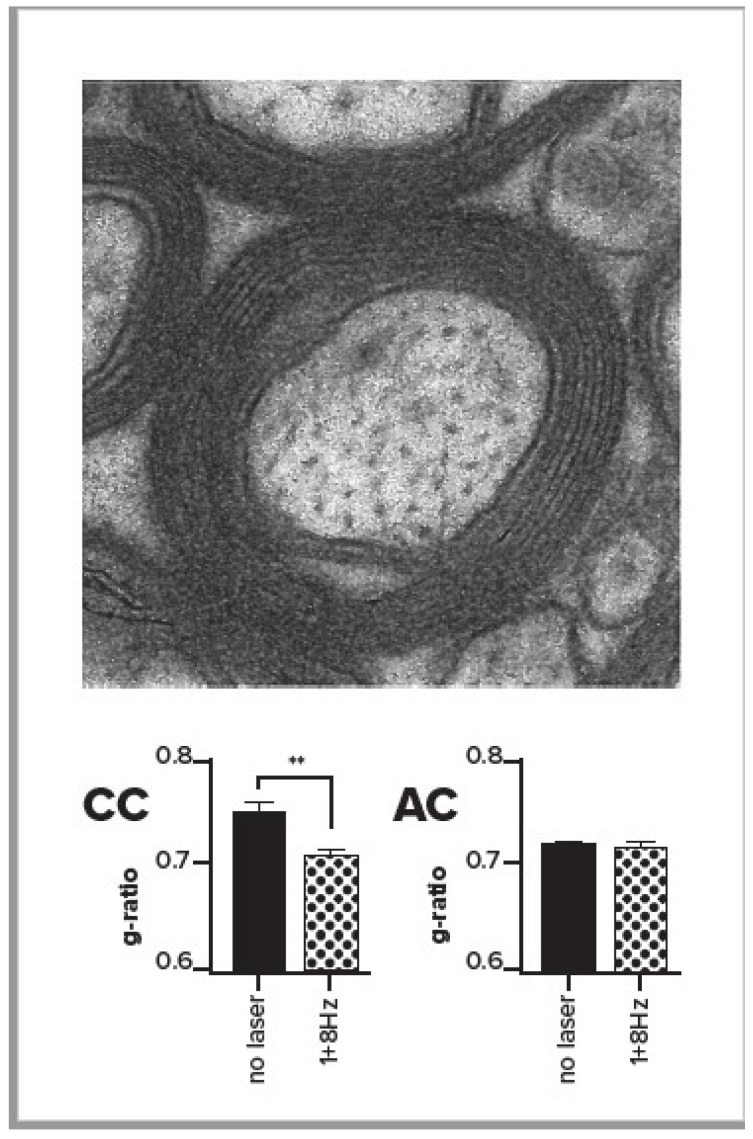
Top of figure shows an electron micrograph centered on a single axon with myelin rings. Bottom bar graphs show the g-ratio (axon diameter/axon diameter + myelin) in stimulated and unstimulated mice in the corpus callosum close to the site of stimulation (CC, left) or anterior commissure far from stimulation (AC, right) From Piscopo et al. [32] with permission of author and publisher.

We measured the effects of stimulation in the region of corpus callosum that contains output axons from ACC. We found that when the output of the ACC was increased by rhythmic stimulation in the range of 1–8 Hz, there was an increase in oligodendrocytes [32], the cells that myelinate axons. This effect was significant with 1 Hz, in the same direction but not significant with 8 Hz, and reversed with the 40 Hz rhythm. We also used electron microscopy (EM, see Figure 3) to directly measure axonal myelination at the ultrastructural level [32] in ten mice, six of which received low frequency (1 or 8 Hz stimulation), and four of which were unstimulated controls. We compared the g-ratio (axonal diameter/axonal diameter + myelin) in stimulated versus unstimulated mice and found that the g-ratio was significantly reduced in the corpus callosum for stimulated mice in comparison with unstimulated controls (see bottom left panel of Figure 3). Furthermore, the g-ratio was unchanged in the anterior commissure, which, unlike the corpus callosum, does not contain axons from ACC (Figure 3, bottom right). The number of observations was too small to provide a test of whether 1 or 8 Hz might be the most effective. The reduced g-ratio in the ACC for the stimulated mice reflected the greater myelin as well as reduced axonal diameter in comparison with controls.

We also found a behavioral effect of low frequency stimulation, particularly in the 8 Hz group, using a light/dark assay where more time in the dark is used as a measure of anxiety. We found that time spent in the light when given a choice between light and dark areas of the cage was increased in those mice in the low frequency stimulation group, compared to unstimulated controls [31], demonstrating increased exploration and suggesting decreased anxiety.

## 6. Optogenetics and Orienting

It now seems possible to use optogenetic methods to determine which networks are necessary in carrying out an orienting task in mice. For instance, a recent study used optogenetic manipulation of the thalamic reticular nucleus to study shifting attention across sensory modalities (auditory vs. visual) [33], and a similar approach could be used to study cued orienting of attention, particularly in a task such as Wang and Krauzlis [10] described above.

Ideally, such studies would target the specific pathways and networks found to be involved in orienting in human studies, such as magnocellular versus parvocellular pathways, and areas such as the superior parietal lobe, temporal parietal junction, and the ventral stream pathway, although it is not yet clear how/if these map onto the mouse brain’s organization. There is not a clearly layered organization of magnocellular and parvocellular pathways in the mouse lateral geniculate nucleus (LGN), although there are multiple response types including transient and sustained cells that may correspond [34]. Anatomical and functional evidence does suggest a rough segregation of the mouse extrastriate cortex into dorsal and ventral pathways [35,36], so manipulating specific extrastriate areas may provide a more direct comparison of networks across species.

In combination with optogenetic manipulation of key areas of these networks, one could use a variety of cues to orient the animals to the target location prior to its onset. If the laser suppresses a particular area during the cue, there should be a reduction in the ability of the mice to orient to the cue and, thus, slower reaction time to targets at the cued location. One could compare symmetric circles and squares with asymmetric arrows after subjects have learned to associate the cue with a location. Of course, preliminary studies would have to determine whether they can discriminate these shapes, and whether they could learn to associate the cue properties (such as arrow direction) with a location. If the Lambert theory [6] is correct, optogenetic inactivation within the “what” pathway, such as the lateral extrastriate areas described by Wang et al. [35], should interfere with all forms of symmetric symbolic cues whether or not they were word- or letter-like. If, however, parietal areas are important for the orienting response with symbolic cues, the “what” pathway inhibition should have little or no influence on reaction time. These mouse studies would not dispute the finding by Lambert et al. [6] that letter-like stimuli use the “what” pathway, since animals would not have the visual word form system, but they would allow a more detailed analysis of the role of various parietal pathways in the orienting of attention.

It might also be possible to use the mouse to explore more naturalistic forms of orienting. Recent studies have demonstrated that mice use vision in prey capture, to orient toward, approach, and capture crickets [37]. Based on studies in other species, this behavior likely depends on the superior colliculus, providing a connection between overt orienting of motor output and orienting of attention [38]. Studying the cell-type specific circuits involved in this prey capture paradigm might allow the exploration of orienting mechanisms in ecologically valid situations.

One would also like to test which cells are involved in different forms of orienting. For some circuits, this is possible due to the availability of specific Cre driver lines to access distinct cell types, such as in superior colliculus [39]. Unfortunately, there are not yet specific driver lines for pathways in the mouse LGN and there are not distinct layers, so it is not currently possible to manipulate them separately, although future developments may allow this. However, work in the monkey [40], where the magnocellular and parvocellular pathways are well separated, shows that optogenetic studies could be used to inhibit them and determine their involvement in various forms of orienting of attention.

## 7. Conclusions

Orienting of attention has been studied in both humans and animals, and these studies have begun to show parallels in the mechanisms and pathways involved across species. Recent findings have proposed several networks that might be engaged to control covert orienting to cues. We expect that optogenetics and related methods could be used in behavioral tasks for mice to discriminate among these networks and to test ideas about the specific cell types involved.
